# On the quality evaluation of scientific entities in Poland supported by consistency-driven pairwise comparisons method

**DOI:** 10.1007/s11192-014-1258-y

**Published:** 2014-03-19

**Authors:** Waldemar W. Koczkodaj, Konrad Kułakowski, Antoni Ligęza

**Affiliations:** 1 Laurentian University, Sudbury, ON Canada; 2 AGH University of Science and Technology, Kraków, Poland

**Keywords:** Pairwise comparisons, Inconsistency analysis, Expert opinion, Academic entity quality, Performance evaluation

## Abstract

Comparison, rating, and ranking of alternative solutions, in case of multicriteria evaluations, have been an eternal focus of operations research and optimization theory. There exist numerous approaches at practical solving the multicriteria ranking problem. The recent focus of interest in this domain was the event of parametric evaluation of research entities in Poland. The principal methodology was based on pairwise comparisons. For each single comparison, four criteria have been used. One of the controversial points of the assumed approach was that the weights of these criteria were arbitrary. The main focus of this study is to put forward a theoretically justified way of extracting weights from the opinions of domain experts. Theoretical bases for the whole procedure are based on a survey and its experimental results. Discussion and comparison of the two resulting sets of weights and the computed inconsistency indicator are discussed.

## Introduction and problem statement

The question of how to measure the performance of scientific entities is one of the most basic in the scientific community. The answer to this question is primarily related to: ’how should research funds be distributed among different research units?’ (addressed in Wang et al. 2011; Geuna and Martin 2003), or ’what should be the policy of the state in the promotion of science?’ (see Geuna et al. 1999) to name a few. Due to the many factors that can affect the final assessment, the problem of finding clear and widely acceptable performance indicators is not easy. Numerous legal environments and various scientific practices in different countries add to the problem complexity.

In academia, we are better in evaluating our students than ourselves. However, the Ministry of Science and Higher Education stipulates that evaluation of the academic performance of a scientific unit 1 is conducted in Poland on the basis of the algorithm presented in the government regulation 2 with four major criteria for all scientific entities (Table 1).

**Table 1:** Comparison criteria for scientific entities

Code	Criterion name
*c*_1_	Scientific and/or creative achievements
*c*_2_	Scientific potentiality
*c*_3_	Tangible benefits of the scientific activity
*c*_4_	Intangible benefits of the scientific activity

Each criterion is subdivided into many sub-criteria that depend on the type of scientific entity. The ranking process seems to be easy, yet it is not. One of the important problems is to determine the significance of criteria $c_{1},\ldots,c_{4}$. Relating *c*_*i*_ to *c*_*j*_ is both subjective and difficult due to the intangible and abstract nature of the criterion itself. In the adopted algorithm (“Research entities evaluation: The official procedure” Section), the criteria importance must be expressed as the real numbers. One of the ways allowing the subjective judgments to be transformed into the numerical values is the pairwise comparisons (PC) method. Therefore, to improve the algorithm proposed by the Ministry of Science and Higher Education (“Research entities evaluation: The official procedure” Section), the authors propose to add one additional step. The step in which the weights for criteria $c_{1},\ldots,c_{4}$ are explicitly estimated by experts. The experiment conducted by the authors (“An experimental survey procedure” Section), the survey among the scientists, provides a sample of how the weights of the criteria $c_{1},\ldots,c_{4}$ might look like, when they were determined by the PC method.

## Preliminaries of the PC method

As indicated in “Introduction and problem statement” Section, the evaluation of research units and induction of the final linear ordering is based on four different criteria. These predefined criteria are shown in Table 1. Precise interpretation of these criteria is provided by the Ministry of Science and Higher Education (2012); here the intuitive understanding of them is sufficient.

Note that in view of Table 1 quality evaluation of research units is not only a Multicriteria Decision Problem (or, more precisely, Multicriteria Ranking Problem), but all the criteria are in fact of *qualitative nature*. Hence, the first step in the procedure consists of defining the transformation of non-measurable characteristics into a single numbers. This is done for each criterion of each research unit. For example, calculation of the value of criterion *c*_1_ consists in summing up points assigned to a list of publications of the last four years published by the research workers of the unit. The procedure is presented in detail in Ministry of Science and Higher Education (2012). Now, the problem can be approached by pairwise comparisons.

Let us briefly review the roots and ideas of the approach. It is believed that, in 1785, Condorcet was the first researcher who used pairwise comparisons for improving voting results in Condercet (1785). However, it was Fechner who described the *PC* method in in 1860 (reprinted in Fechner 1966), but he did it only from the psychometric perspective. Thurstone not only described the PC method in Thurstone (1994), but for the first time proposed a solution based on statistical analysis. In his seminal work Saaty (1977), Saaty introduced a hierarchy, which is instrumental for practical applications, and eigenvalue-based inconsistency.

Regretfully, the proposal of Saaty constitutes only a global inconsistency indicator and, as such, could not localize the most inconsistent elements of the matrix. The first ever localizing inconsistency definition was proposed in Koczkodaj (1993). Both inconsistencies were recently analyzed in Bozóki and Rapcsak (2008).

There are several different ways for deriving weights in the pairwise comparisons method Crawford (1987); Kułakowski (2013). For the purpose of this paper, the authors adopted probably the second most popular geometric means based method. The Monte Carlo study presented in Herman and Koczkodaj (1996) provided evidence that, for small inconsistencies, both the geometric means solution (used in this study) and the eigenvector solution (as proposed by Saaty in 1977) are similar enough from the statistical point of view. In fact, eigenvector and geometric means solutions are identical for fully consistent matrices and the geometric means is slightly better (approx. 7 out of 10 wins) than the principal eigenvector solution.

The procedure usually begins (after an appropriate feasibility study and data gathering, which are not addressed here) with a listing of all possible criteria. In our case, the four criteria mentioned in “Preliminaries of the PC method” Section are used.

Let us consider *n* alternative items to be compared. Let *m*_*ij*_ expresses a relative preference of entity *s*_*i*_ over $s_{j}, i,j=1,\ldots,n$. The pairwise comparison matrix *M* is defined as

1 $$ M=\left[ \begin{array}{llll} 1 &m_{12} & \cdots & m_{1n}\\ \frac{1}{m_{12}} & 1 & \cdots & m_{2n}\\ \vdots & \vdots & \vdots & \vdots\\ \frac{1}{m_{1n}} & \frac{1}{m_{2n}} & \cdots & 1 \end{array}\right] $$

A pairwise comparisons matrix *M* describing the relationship between *n* given alternative items is called *reciprocal* if $m_{ij}=\frac{1}{m_{ji}}$ for every $i,j=1,\ldots,n$ (then automatically *m*_*ii*_ = 1 for every $i=1,\ldots,n$). Let we say that $M=[m_{ij}]\in R^{n\times n}$ is a pairwise comparisons (PC) matrix if *m*_*ij*_ > 0 for all *i*, *j* = 1, ..., *n*. A PC matrix *M* is called *consistent* (or transitive) if $m_{ij}\cdot m_{jk}=m_{ik}$ for every $i,j,k=1,\ldots,n$. Note that while every consistent matrix is reciprocal, the converse is false in general. Consistent matrices correspond to the ideal situation in which there are the exact values $s_{1},\ldots,s_{n}$ for the entity. The elements of matrix *M* defined as quotients *m*_*ij*_ = *s*_*i*_/*s*_*j*_ form a consistent matrix. The vector $\mathbf{s}=[s_{1},\ldots,s_{n}]$ is unique up to a multiplicative constant.

Every pairwise comparisons question has been answered by all respondents and many different pairwise comparisons matrices could be produced (one matrix for each expert involved in the survey). In our study, we produced the survey summary for all the partial results $M_{1},\ldots,M_{q}$ (every *M*_*r*_ = [*m*_*ij*_^(*r*)^] corresponding to responses given by the i’th expert) by synthesizing them into one summary PC matrix $\widehat{M}=[\widehat{m}_{ij}]$ following the geometric mean synthesizing function proposed in Aczél and Saaty (1983); Saaty (2008) where all the experts are equally important.^3^ Hence, the resulting ratios have the following form:

2 $$ \widehat{m}_{ij}=\left(\prod_{r=1}^{q}m_{ij}^{(r)}\right)^{{1/q}} $$

According to the geometric mean method used in this study the final ranking vector *s* is calculated as:

3 $$ s=[\sigma^{-1}S_{1},\ldots,\sigma^{-1}S_{n}] $$

where

4 $$ S_{i}=\left(\prod_{j=1}^{n}\widehat{m}_{ij}\right)^{{1/n}} \hbox{ and  }\quad \sigma=\overset{n}{\underset{r=1}{\sum}}S_{r} $$

In the formulas above *S*_*i*_ represents the rank of the *i*-th alternative before normalization, and σ^−1^ is the normalization coefficient so that all *s*_*i*_ = σ^−1^ *S*_*i*_ for $i=1,\ldots,n$, sum up to one.

Let us see how the pairwise comparisons method works in practice by providing the following simple numeric example in which four widely recognized scientists apply for some award for their research results. For simplicity let us assume that the assessment procedure is based on an review of a few major scientific achievements of each. During the evaluation panel two experts provide their assessments forming two matrices *M*_1_ and *M*_2_.

5 $$ M_{1}=\left[ \begin{array}{llll} 1 &0.4 & 1.1 & 0.91\\ 1/0.4 & 1 & 1.28 & 1.1\\ 1/1.1 & 1/1.28 & 1 & 0.5\\ 1/0.91 & 1/1.1 & 1/0.5 & 1 \end{array}\right] \quad M_{2}=\left[ \begin{array}{llll} 1 & 0.5 & 1.3 & 0.85\\ 1/0.5 & 1 & 1.28 & 1.2\\ 1/1.3 & 1/1.28 & 1 & 0.5\\ 1/0.85 & 1/1.2 & 1/0.5 & 1 \end{array}\right] $$

To determine the PC matrix, each expert had to perform six comparisons (ones corresponding to the values above the matrix diagonal). For example, by indicating that the achievements of the first candidate are 0.4 of the achievements of the second candidate *m*_1,2_^(1)^ = 0.4 the first expert indicated that *m*_2,1_^(1)^ = 1/0.4 = 2.5. Thus, the values below the diagonal in *M* ^(1)^ and *M* ^(2)^ are generated in an automatic way. The matrices *M* ^(1)^ are used *M* ^(2)^ to compute the matrix of collective results $\widehat{M}$ according to the formula (Eq. 2).

6 $$ \widehat{M}= \left[\begin{array}{llll} 1 &0.447 & 1.196 & 0.879\\ 2.236 & 1 & 1.28 & 1.149\\ 0.836 & 0.781 & 1 & 0.5\\ 1.137 & 0.87 & 2 & 1 \end{array}\right] $$

The final assessment **s** is formed as the normalized geometric means of rows of $\widehat{M}$ and is **s** = [0.201, 0.327, 0.184, 0.288]^*T*^. Hence, the winner is the second scientist with the rank 0.327, then respectively the scientist number four, one and three.

## Data inconsistency and how to deal with it

Observe that matrix $M^{\star}$ given by (5) is not consistent. For example $m_{1,2}^{\star}\cdot m_{2,3}^{\star}\neq m_{1,3}^{\star}$ as $0.4\cdot1.1\neq0.5$. The question arises what can one do about that?

When an *n* × *n* matrix *M* is not consistent the consistency index needs to be computed to determine the degree of inconsistency. One of the popular inconsistency index Saaty (1977) is defined as follows:

7 $$ Ic(M)=\frac{\lambda_{max}-n}{n-1} $$

where λ_*max*_ is the principal eigenvalue of *M*. It is commonly assumed that the matrix *M* is sufficiently consistent if *Ic*(*M*) ≤ 0.1 Saaty (1977). In such a case the results calculated using e.g. the geometric means method are considered to be reliable.

Another approach, perceived as more restrictive inconsistency index comes from Koczkodaj (1993). It is defined as:

8 $$ {\fancyscript{K}}(M)=\underset{i,j,k\in\{1,\ldots,n\}}{\max}\left\{ \min\left\{ \left|1-\frac{m_{ij}}{m_{ik}m_{kj}}\right|,\left|1-\frac{m_{ik}m_{kj}}{m_{ij}}\right|\right\} \right\} $$

where $i,j,k=1,\ldots,n$ and *i* ≠ *j*∧ *j* ≠ *k*∧ *i* ≠ *k*. For sufficiently consistent matrices, it should not be too high.

When a matrix *M* is inconsistent (especially when the inconsistency is high), we must compute a consistent *n* × *n* PC matrix *C* which differs from the matrix *M* ’as little as possible’. This is a relatively simple and natural way of dealing with the problem. Note that the approximation is really reduced to a problem of norm selection and the distance minimization. For the Euclidean norm, the vector of geometric means (equal to the principal eigenvector for the transitive matrix) is the one which generates it.

Many approximation solutions have been proposed in the past starting with Jensen (1984). More recently, Bozóki et al. (2010), and others Anholcer et al. (2010); Grzybowski (2012) proposed a practical optimization. No study has ever provided an analytic proof of the substantial superiority of any method for approximation over another. Strong statistical evidence (based on 1,000,000 randomly generated matrices) suggests that both solutions (geometric means and the principal eigenvector) are reasonable and do not differ much for ’not-so-inconsistent’ (NSI) matrices, as demonstrated in Herman and Koczkodaj (1996).

A further investigation of the selection of the norm (or distance) is beyond the scope of this study. In fact, it may require many years of research before any conclusions could be made and probably the pairwise comparisons may be helpful in it. Unfortunately, not much can be analytically proven for non-transitive matrices. In data processing, it is well expressed by the popular computer concept GIGO (Garbage In—Garbage Out). GIGO summarizes what is known for a long time: getting good results from ’dirty data’ is unrealistic and certainly cannot be guaranteed.

## Research entities evaluation: the official procedure

The evaluation procedure officially adopted in *Poland* for assessment of research units consists of six steps. Some of them are more or less informal and based largely on the work of experts, whilst the other ones are precisely defined with extensive use of mathematical formulas. In particular the final results of the algorithm highly depends on subjectively defined weights $W_{1},\ldots,W_{4}$ describing importance of each of the criteria $c_{1},\ldots,c_{4}$, as presented in “Preliminaries of the PC method” Section.

Note that due to diversification of the research activities in different areas of science, all the units are divided into relatively small groups of similar entities (e.g. Faculties of Electrical Engineering). Hence, all the 963 units were divided into similarity groups (GWO) of a limited number of units (for example, around 50 in a typical GWO). The procedure was performed independently for each group.

### Assessment procedure

1. At the beginning experts proposed weights $W_{1},\ldots,W_{4}$ for each group of mutually comparable entities.
2. Then, each scientific entity *X* is assigned numerical values with respect of the four criteria as defined in (Table 1). As a result, a vector of four values $O_{1}(X),\ldots,O_{4}(X)$ defining how good is unit *X* with respect to $c_{1},\ldots,c_{4}$ is *X* is prepared.
3. The experts proposed two artificial entities *A*_1_ and *A*_2_ which will be used as reference units in order to assign every real research unit an appropriate funding level. *A*_1_ and *A*_2_ become part of a ranked group.
4. All the entities are mutually compared within its GWO of comparable entities with respect to all four criteria (Table 1). The result of a single comparison of $X,Y\in U$, where *U* is the GWO for *X* and *Y*, with respect to the *i*-th criterion is given as:
  9 $$ P_{i}(X,Y)=sgn(O_{i}(X)-O_{i}(Y))\cdot \left\{ \begin{array}{ll} 0 & \hbox {if} \quad \Updelta O<D\\ \frac{\Updelta O-D}{G-D} & \hbox {if} \quad D\leq\Updelta O<G\\ 1 & \hbox {if} \quad G\leq\Updelta O\\ \end{array}\right. $$
  where
  10 $$ \Updelta O=\left|O_{i}(X)-O_{i}(Y)\right| $$
  11 $$ D=max\left\{ \frac{min\left\{ O_{i}(X),O_{i}(Y)\right\} }{10},\frac{\sum_{Z\in U}O_{i}(Z)}{10\cdot card(U)}\right\} $$
  12 $$ G=\max\left\{ \frac{3\cdot\min\left\{ O_{i}(X),O_{i}(Y)\right\} }{10},3\cdot D\right\} $$
5. During the currently adopted ranking procedure by the Ministry of Science and Higher Education (2012), the value *V*(*X*, *Y*) is computed according to the formula:
  13 $$ V(X,Y)=\underset{i=1,\ldots,4}{\sum}W_{i}P_{i}(X,Y) $$
  where *V*(*X*, *Y*) is the total comparison score of the scientific unit *X* versus *Y*, *W*_*i*_ is the rank (importance) of the *i*-th criterion, and *P*_*i*_(*X*, *Y*) is the result of the pairwise comparisons between *X* and *Y* with respect to the *i*-th criterion.
6. The final rank of the scientific entity $X\in U$ is computed as:
  14 $$ R(X)=\frac{1}{card(U)-1}\left(\underset{Y\in U\backslash\{X\}}{\sum}V(X,Y)\right) $$
  where $U=\{X_{1},\ldots,X_{card(U)}\}$ is the set of the scientific and the two reference (artificial) units to be assessed.

### Numerical example

To better understand the algorithm, let us assume that *U* for some specific type of scientific entities (GWO) consists of six elements $U=\{X_{1},\ldots,X_{4},A_{1},A_{2}\}$. The result vectors of every scientific unit including the two referential ones determined by experts 4 are given in (Table 2).

**Table 2:** Group of four mutually comparable scientific entities

Id.	Entity name	*O*_1_	*O*_2_	*O*_3_	*O*_4_
1	*X*_1_	51.97	525	12.64	84.5
2	*X*_2_	84.07	127	1.02	20
3	*X*_3_	41.11	583	4.22	88
4	*X*_4_	33.79	246	7.21	60
5	*A*_1_	39.07	455.40	12.12	59.76
6	*A*_2_	18.99	221.37	5.89	29.05

The original weights as proposed for this comparisons group in the original algorithm are *W*_1_ = 0.65, *W*_2_ = 0.1, *W*_3_ = 0.15 and *W*_4_ = 0.1.

To determine the ranking value *R*(*X*_1_) every $V(X_{1},X_{2}),\ldots,V(X_{1},X_{4}), V(X_{1},A_{1})$ and *V*(*X*_1_,*A*_2_) need to be computed, then the average according to (Eq. 14) need to be computed. For example, to determine *V*(*X*_1_,*X*_3_) every single *P*_*i*_(*X*_1_,*X*_3_) need to be calculated. Thus, following the procedure (“Assessment procedure” Section) it is easy to see that the comparisons are *P*_1_(*X*_1_, *X*_3_) = 0.711, *P*_2_(*X*_1_, *X*_3_) =  − 0.052, *P*_3_(*X*_1_, *X*_3_) = 1 and *P*_4_(*X*_1_,*X*_3_) = 0. Since, the rank value of *X*_1_ and *X*_3_ is defined as

15 $$ V(X_{1},X_{3})=W_{1}P_{1}(X_{1},X_{3})+\cdots+W_{4}P_{4}(X_{1},X_{3}) $$

hence we have $V(X_{1},X_{3})=0.65\cdot0.711-0.1\cdot0.052+1\cdot0.15+0=0.607$, and, simultaneously, *V*(*X*_3_,*X*_1_) =  − 0.607.

After consecutive repeating the procedure for every pair it can be calculated that *V*(*X*_1_, *X*_2_) =  − 0.3, *V*(*X*_1_, *X*_4_) = 1, *V*(*X*_1_, *A*_1_) = 0.736 and *V*(*X*_1_,*A*_2_) = 1. Thus, the final score for *X*_1_ is $R(X_{1})=\frac{1}{5}(-0.3+0.607+1+0.736+1)=0.609$. After calculating *R* for all other entities algorithm stops. The obtained rank is as follows: *R*(*X*_1_) = 0.609, *R*(*X*_2_) = 0.318, *R*(*A*_1_) = 0.046, *R*(*X*_3_) = 0.028, *R*(*X*_4_) =  − 0.21 and *R*(*A*_2_) =  − 0.791.

The final results of the evaluation procedure are given by the following linear ordering: *X*_1_, *X*_2_, *A*_1_, *X*_3_, *X*_4_, *A*_2_. Since there are two referential units, apart of the linear ordering the units are assigned to three categories: A—for the leading ones (here: *X*_1_,*X*_2_, B—for the medium class (here: *X*_3_,*X*_4_), and C—for ones which must improve (here: empty).

## The need for a better method for the weight selection

Needless to say that the weights $W_{1},\ldots,W_{4}$ (see step 5 of the procedure) have a significant influence on the final results of the ranking process. Their values determine what kind of achievements (and to what extent) are preferred. Hence, the choice of these weights determines the required policy of the development of scientific entities in *Poland* (the rank position translates into an appropriate funding level).

Recall that these weights were defined by experts in an arbitrary way. Due to the significance of their values, we propose to adopt the selection procedure by computing values of the weighting coefficients from their pairwise comparisons.

There are several reasons to this approach be considered acceptable. One of them is intangibility of the compared achievement assigned to each of the evaluation criteria. Tangible things can be easily measured with reference to some specific unit. Thus, the measure determines the levels of desired features. The intangible factors can be compared in pairs Saaty (2013) without an a priori measurement. In the domain literature, there is considerable evidence indicating that the pairwise comparisons method works when the intangible objects need to be compared Subramanian and Ramanathan (2012).

The algorithm criteria as mentioned in (Table 1) reflect intangible achievements. Experts need a method that would allow them to assess all the objects. The pairwise comparisons method simplifies it by reducing the comapred objects to only two at a time.

Note that the weights tuning mechanism is used also in AHP (Analytic hierarchy process)—another decision making scheme based on the comparing objects in pairs Saaty (1977). In the context of the *AHP* method such weights are often referred to as the criteria with respect to the goal evaluation. Of course, the *AHP* uses the weights in a bit different way. However, the regularity that, the higher the weight of the criterion is, the greater is its impact on the final result, is preserved.

Selection of such important factors as weights in the scientific entity evaluation procedure should be based on transparent, well justified mechanisms. The results should gain a widespread acceptance among the members of the evaluated units. Here again, the pairwise comparisons method can be helpful.

As it is shown in the experiment (see “An experimental survey procedure” Section in the evaluation process may attend any number of experts from different research centers. The pairwise comparisons method also addresses the inconsistency problem. It allows the experts to measure Saaty (1977), to localize Koczkodaj (1993) and to reduce Koczkodaj and Szarek (2010) the inconsistency of the results of comparisons in pairs.

## An experimental survey procedure

As with any anticipated change, the proposed CERU 5 conceptual model of the assessment process was vigorously debated in the scientific community (see Kistryn 2013). In particular, the weights $W_{1},\ldots,W_{4}$ corresponding to the importance of the criteria $c_{1},\ldots,c_{4}$ (Table 1) were the subject of debate and criticism since they were established in an arbitrary way.

The goal of the experimental survey was to provide the weighting coefficients assuming that:

- the PC method is the core of the experiment; so the values are better justified,
- any arbitrarily large number of experts can express their preferences,
- the expert judgment consistency should be evaluated and kept at a possible low level.

The relative preference criteria are established on the basis of partial values (pairwise comparisons) provided by experts in form of the matrix *M* (see Eq. 1). All the matrices for which inconsistency index (Eq. 7) is higher than 0.1 are excluded from the ranking as not reliable enough.

The final weights are computed according to the PC methodology given as (Eq. 3). The authors invited members of the academic community who know the specificities of Polish research units to provide expert assessments by an Internet survey. The surveyed scientists used the reference scale (Table 3) that helps them in translating intuitive meanings of assessments into numbers.

**Table 3:** Comparison scale—the values 1,2 and 3 are assigned to the appropriate definitions of intensity or importance. Intermediate judgments are also possible. E.g. value 1.4 corresponds to the situation when one criterion is slightly more preferred than the other

Value	Definition of intensity or importance	Explanation
1	Equal importance	Two criteria equally contribute to the objective
2	Essential or strong importance	Experience and judgments favor one criterion over another
3	Absolute importance	The highest affirmation degree of favoring one criterion over another
1.4	Intermediate judgments	An expert prefers slightly one criterion over another

The choice of scale is also a challenging problem. It has been extensively discussed in the literature Fülöp et al. (2010); Dong et al (2008); Ji and Jiang (2003); Salo and Hämäläinen (1997); Triantaphyllou et al. (1994). There is no “one fits all” scale, although some studies argue that a certain scale should give more reliable results than another. In Fülöp et al. (2010), a small scale from 1 to 3 (Table 3) is shown to have the best mathematical properties (relted to the convexity)for the PC method. It was adopted by the authors for this study. After all, practically all modern languages have only three levels of gradation in the grammar (e.g., good -> better -> the best).

Despite the scale recommendation the Internet survey application allowed respondents to set any value (except 0) of the *m*_*ij*_ ratio between $\frac{1}{99}$ and 99. Introduction (suggestion) the scale while allowing almost the free choice of ratio is an attempt to find a compromise between the desire to give an intuitive interpretation for some numerical values (the scale), and allowing the experts to the greatest possible precision in expressing beliefs. Moreover, thanks to introducing the scale all the experts share the same correspondence between the numerical values and the intuitive descriptions of importance. This helps to minimize the risk of situation in which two experts sharing the belief that *c*_*i*_ is absolutely more important than *c*_*j*_ assign two essentially different (although greater than one) values of *m*_*ij*_. The scale introduction allows for identification all such cases, and excluding the identified outliers from the ranking. The candidates for outliers are experts whose answers are significantly off the scale. Usually their response also has a large inconsistency.

The meaning of the adopted scale is quite intuitive. For example, if an expert assigns *W*_*i*_/*W*_*j*_ to 1, this means that the criteria *i* and *j* are of equal importance. On the other hand if, for instance, 2 < *W*_*i*_/*W*_*j*_ < 3 then, according to the adopted textual interpretation (Table 3), the *i*-th criterion was recognized as essentially more important than the *j*-th one.

In the ideal case, there should be always ${W_{i}/W_{j}}\cdot{W_{j}/W_{k}}={W_{i}/W_{k}}$. However, because each of the three ratios are determined independently, in practice this is often not the case. Hence, very often there are some triads of ratios which do not meet this equality. This situation is related to the problem of data inconsistency in the PC matrix, which is discussed more thoroughly in “Data inconsistency and how to deal with it” Section.

## Survey results

### Survey data

The survey involved *37* researchers from *17* Polish and foreign scientific institutions engaged in research in the field of technical and engineering sciences. Most of them are tenured faculty members at Universities in Poland, USA, Canada, and Australia although some of them declared employment in research institutes. The vast majority of respondents declared the position of a full professor or equivalent.^6^ A few persons held the prestigious title of distinguished professor.

Every participant of the survey had to answer six questions and, thus, determine six ratios: $\frac{w_{1}}{w_{2}},\frac{w_{1}}{w_{3}},\frac{w_{1}}{w_{4}},\frac{w_{2}}{w_{3}},\frac{w_{2}}{w_{4}},\frac{w_{3}}{w_{4}}$. The answers allowed for formation of the partial *PC* matrix *M*_*r*_ in the form:

16 $$ M_{r}= \left[ \begin{array}{llll} 1 &\frac{w_{1}}{w_{2}} & \frac{w_{1}}{w_{3}} & \frac{w_{1}}{w_{4}}\\ \frac{w_{2}}{w_{1}} & 1 & \frac{w_{2}}{w_{3}} & \frac{w_{2}}{w_{4}}\\ \frac{w_{3}}{w_{1}} & \frac{w_{3}}{w_{2}} & 1 & \frac{w_{3}}{w_{4}}\\ \frac{w_{4}}{w_{1}} & \frac{w_{4}}{w_{2}} & \frac{w_{4}}{w_{3}} & 1 \end{array}\right] $$

To synthesize the final results the authors used almost all the gathered matrices *M*_*r*_. The only exceptions were five result sets with the very high inconsistency index ${\fancyscript{K}(M_{r})}$ (over 0.836), and the inconsistency index *Ic*(*M*_*r*_) higher than 0.1. Although all the rejected cases differ in detail, most of the rejected authors indicated very significant importance of the first criterion (scientific and/or creative achievements) over other arbitrarily chosen criteria. Unfortunately, due to the large inconsistency (in the literature *Ic*(*M*_*r*_) higher than 0.1 is considered as unacceptable Saaty 2005) their opinions have not been taken into account 7 in the synthesized matrix $\widehat{M}$.

All the 32 admissible partial results *M* form the following final output matrix $\widehat{M}$, which looks as follows:

17 $$ \widehat{M}= \left[ \begin{array}{llll} 1 &1.813 & 1.503 & 1.784\\ 0.552 & 1 & 0.952 & 1.296\\ 0.666 & 1.05 & 1 & 1.302\\ 0.561 & 0.772 & 0.768 & 1 \end{array} \right] $$

The normalized weight vector ω derived from $\widehat{M}$ using the geometric mean method is as follows:

18 $$ \omega= \left[ \begin{array}{llll} 0.36 &0.22 & 0.236 & 0.184 \end{array} \right]^{T} $$

which means that the invited experts found that *rank*(*c*_1_)—the relative importance of *scientific and/or creative achievements* criterion is 0.36, *rank*(*c*_2_)—*scientific potentiality* criterion is 0.22, *rank*(*c*_3_)—*tangible benefits of the scientific activity* criterion is 0.236, and finally *rank*(*c*_4_)—*intangible benefits of the scientific activity* criterion is 0.184.

The inconsistency indices for $\widehat{M}$ are low. The more sensitive for local perturbations Koczkodaj’s index ${\fancyscript{K}(\widehat{M})=0.241}$ whilst $Ic(\widehat{M})=0.002$. The standard geometric deviation for the appropriate $\widehat{m}_{ij}$ defined as:

19 $$ \sigma_{g}(\widehat{m}_{ij})=\exp\left(\sqrt{\frac{\sum_{k=1}^{32}\left(\ln m_{ij}^{(k)}-\ln\widehat{m}_{ij}\right)^{2}}{32}}\right) $$

forms the matrix $\widehat{M}_{\sigma}=[\sigma_{g}(\widehat{m}_{ij})]$ as follows:

20 $$ \widehat{M}_{\sigma}= \left[ \begin{array}{llll} 1 &2.093 & 2.03 & 1.787\\ 2.093 & 1 & 1.913 & 1.831\\ 2.03 & 1.913 & 1 & 1.61\\ 1.787 & 1.831 & 1.61 & 1 \end{array} \right] $$

It is easy to see (Eq. 20) that the most controversial (with the highest standard geometric deviation) comparison is between the *scientific and/or creative achievements* *c*_1_, and the *scientific* *potentiality* *c*_2_. On the other hand experts were most unanimous comparing *c*_3_ and *c*_4_ (the standard geometric deviation of $\sigma_{g}(\widehat{m}_{34})=\sigma_{g}(\widehat{m}_{43})=1.61$ is the closest to 1).

### Results: different perspectives

**Fig. 1:**
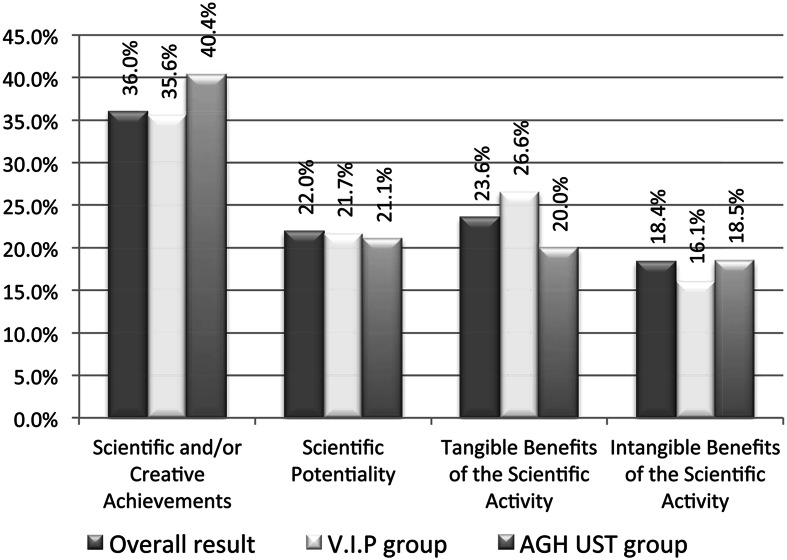
The overall survey results with the VIP and AGH UST employee groups

Experts were chosen at random among those who know the specificity of Polish technical scientific units. Most of the experts are affiliated at the Polish universities or research institutes. Three experts are affiliated at foreign universities, although they worked at Polish universities in the past. Since the aim of the survey was to propose the weights $w_{1},\ldots,w_{4}$ for technical scientific units (including such units as the departments of mathematics, physics or computer science), hence most experts is working or has worked in such institutions. On the other hand, it was important for the authors of the survey that the experts came from different research centers. The best represented university is *AGH UST*—the place of work of the second and the third author. The representations of other *16* scientific units count from one to three experts. Out of the all respondents the authors chose the *VIP* group of six the most influential people consisting of distinguished professors and former or current members of official governmental and scientific bodies, including CERU. The overall results taking into account two special groups: *VIP* group and experts employed at the best represented *AGH UST* are shown below (Fig. 1).

**Fig. 2:**
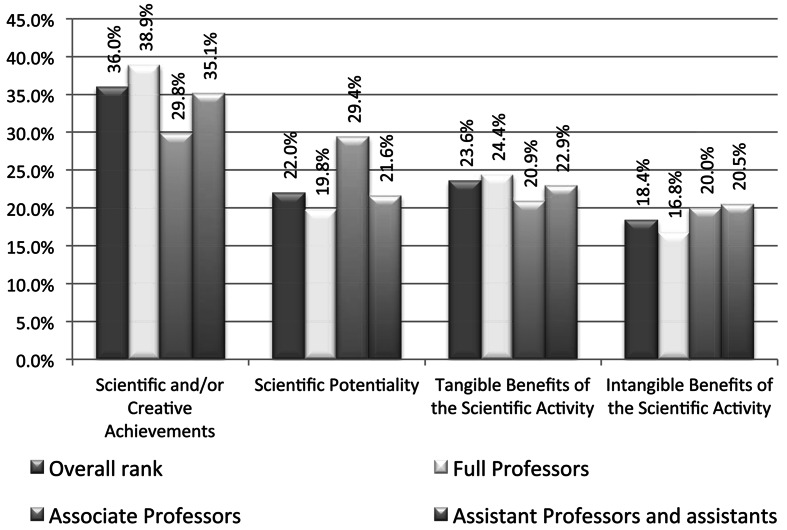
The survey results in groups of full professors, associate professors, and assistant professors and assistants (the overall result was left as the reference)

Among the experts whose opinion have been taken into account there may be distinguished a group of full professors (or professor ordinarius)—18 persons, associate professors (or doctors with habilitation)—6 persons, assistant professors (or doctors)—7 persons, assistants—1 person. The ranking result with respect of these groups (assistant professors and assistants are treated as a single group) are shown below (Fig. 2).

### Montecarlo discrepancy validation

As a validation method for the survey data the authors adopt ten times repeated twofold cross-validation procedure Kohavi (1995). In every repetition the survey sample is randomly split into two disjoint sets $S_{1}=\{M_{1},\ldots,M_{16}\}$ and $S_{2}=\{M_{17},\ldots,M_{32}\}.$ Both groups are used to synthesize matrices $\widehat{M}_{1}$ and $\widehat{M}_{2}$, next two ranking vectors $a=\left[a_{1},\ldots,a_{4}\right]^{T}$ and $b=\left[b_{1},\ldots,b_{4}\right]^{T}$ are computed. The vector *a* is called the reference rank vector, whilst *b* is called the validation rank vector. For each pair of vectors *a* and *b* the discrepancy vector $d=\left[\left|a_{1}-b_{1}\right|,\ldots,\left|a_{4}-b_{4}\right|\right]^{T}$ is computed.

The values $d_{1},\ldots,d_{4}$ are adopted as a measures of fit. They provide information on how much the synthesized ranking values for the criteria $c_{1},\ldots,c_{4}$ provided by the first group of experts differ from the ranking values provided by the second group. Intuitively speaking the adopted procedure simulates the situation where two disjoint group of experts provide two competitive rankings. Then the first ranking is validated by the second one. The validation procedure has been repeated ten times, so there are ten vectors $a^{(1)},\ldots,a^{(10)}$ and ten vectors $b^{(1)},\ldots,b^{(10)}$. The final reference rank *a* ^avg^ and the discrepancy (fit indicator) vector *d* ^avg^ are computed as arithmetic means:

21 $$ a^{{\rm avg}}=\left[\frac{1}{10}\sum_{i=1}^{10}a_{1}^{(i)}, \ldots,\frac{1}{10}\sum_{i=1}^{10}a_{4}^{(i)}\right] $$

and

22 $$ d^{{\rm avg}}=\left[\frac{1}{10}\sum_{i=1}^{10}d_{1}^{(i)}, \ldots,\frac{1}{10}\sum_{i=1}^{10}d_{4}^{(i)}\right] $$

As a result of the conducted experiment, the following numerical values are obtained:

23 $$ a^{{\rm avg}}= \left[ \begin{array}{llll} 0.364& 0.219 & 0.234 & 0.183 \end{array} \right]^{T} $$

24 $$ d^{{\rm avg}}= \left[ \begin{array}{llll} 0.053 & 0.037 & 0.043 & 0.023 \end{array} \right]^{T} $$

It is easy to see, that the obtained rank result is (on average) similar to the overall result of the survey (Eq. 18). In particular both vectors *a*_*avg*_ (Eq. 23) and ω (Eq. 18) propose the same order of criteria importance. Their individual numerical values are also close to each other. The absolute average absolute difference between individual values in vectors *a* ^(*i*)^ and *b* ^(*i*)^ seem to be reasonably small since they are almost an order of magnitude less than the values in *a* ^avg^. They suggest that regardless of the selection of the group criterion *c*_1_ should be the most important one *a*_1_^avg^ − *d*_1_^avg^ > *a*_*i*_^avg^ + *d*_*i*_^avg^. Unfortunately there is no similar guarantee in the case of any other criterion. The values $a_{1}^{{\rm avg}}\pm d_{1}^{{\rm avg}},\ldots,a_{4}^{{\rm avg}}\pm d_{4}^{{\rm avg}}$ indicate the discrepancy intervals in which the weights of criteria $c_{1},\ldots,c_{4}$ established by the competitive team of experts are expected to be found.

The results *a* ^avg^ and *d* ^avg^ (Eqs. 23, 24) were calculated on the assumption that *S*_1_ and *S*_2_ are equal in size. Thus, in our case both of them count 16 elements. Of course when the size of the set of experts is changing the values *a* ^avg^ and *d* ^avg^ may get changed. For example, according to the intuition (confirmed in tests), the smaller set *S*_1_ (and the larger *S*_2_) the higher discrepancies $d_{1}^{{\rm avg}},\ldots,d_{4}^{{\rm avg}}$. For example for $\left|S_{1}\right|=6$ and $\left|S_{2}\right|=26$ the sample reference rank and the discrepancy vectors are:

25 $$ a_{6}^{{\rm avg}}= \left[ \begin{array}{llll} 0.336 & 0.221 & 0.252 & 0.189 \end{array} \right]^{T} $$

26 $$ d_{6}^{{\rm avg}}= \left[ \begin{array}{llll} 0.083& 0.048 & 0.051 & 0.027 \end{array} \right]^{T} $$

The adopted Montecarlo discrepancy validation procedure tries to model a realistic situation in which one group of experts provides one rank, whilst the other group (disjoint with the first one) creates another rank. Both groups call into question the results of its opponent. As demonstrated by the tests carried out when both groups are composed of experts with a similar scientific background the discrepancies might not be to high.

Also the further research on the inconsistency of synthesized PC matrix $\widehat{M}$ seem to be interesting. In particular the relationship between the values of inconsistency indices $Ic(\widehat{M})$ and ${\fancyscript{K}(\widehat{M})}$ and the deviations of the individual expert judgements in matrices $M_{1},\ldots,M_{r}$ need better explanation.

## Discussion

The survey concerned the basic scientific units at universities in the field of technical and engineering sciences. Thus, the gathered results do not apply to social sciences or the arts. The surveyed researchers have made six comparisons between the four criteria $c_{1},\ldots c_{4}$ (Table 1). They could almost freely choose between ratios from $\frac{1}{99}$ to 99, thus indicating which criterion is more (and how much) important. However, a small scale was recommended following the theory proved in Fülöp et al (2010).

Comparing the survey results (Fig. 1) with the weights adopted in the official government regulation Ministry of Science and Higher Education (2012) (they are: *c*_1_—0.65, *c*_2_—0.1, *c*_3_—0.15, and *c*_4_—0.1) it should be noted that they differ in the intensity of preferences, although they tend to be similar with regard to the order of preferences. In both rankings, the criterion designated as most important is *c*_1_ and the second most important criterion is *c*_3_. However, the weight of *c*_1_ resulting from the survey is almost two times less than the one assumed in the regulation. On the other hand, *c*_2_ obtained from the survey is a bit higher than the one adopted in the official document. According to the survey, the criterion *c*_2_ is slightly less important than *c*_3_ but more important than *c*_4_, whilst the regulation assumes that the weights of *c*_2_ and *c*_4_ are the same. In both these cases the weights obtained from the survey are higher than the ones adopted in the regulation.

The regulation retains the dominant criterion *c*_1_, whilst the other criteria are less important. In fact, it is enough for the scientific entity to be strong in *c*_1_ to avoid having to worry about the other criteria. The survey participants were in favor of a more balanced model in which *c*_1_ is still the most important criterion, but is not predominant. They also appreciate the importance of other criteria with particular emphasis to *c*_3_ (tangible benefits of the scientific activity). Hence, in the model proposed by the surveyed researchers the predominant position of only one criterion *c*_1_ has been replaced by the predominant position of the pair (*c*_1_,*c*_*i*_), where *c*_*i*_ is any other criterion out of *c*_2_, *c*_3_, *c*_4_ (please note that the rank of *c*_1_ and the rank of any other criterion is more than 0.5). Therefore, based on the survey results, such a model would be recommended in which the evaluated scientific entity is good in terms of *c*_1_ but is also good in terms of at least one other criterion, *c*_2_, *c*_3_ or *c*_4_. Of course, the appropriate selection of weights will not solve all the problems related to the scientific entity evaluation algorithm. In particular, it does not prevent the “displacement” of good results in the most important category *c*_1_ by outstanding (in the number but not in the quality nor originality of achievements) results in the less important categories.

The present work tackles many problems and can be a starting point for further research in various areas. In particular, although the new algorithm weights deriving in the official scientific units evaluation procedure is proposed, there are also other highly subjective parts of the algorithm where the PC methods might help. One of them is choosing by experts the so-called reference scientific units.

## Conclusions

The identification of major criteria is a key issue for building a conceptual evaluation model. Once it is done, the final weights are computed from the relative pairwise comparisons by synthesizing them. The model demonstrated in this paper has been used in Poland for evaluating scientific entities consistent with the one proposed by the Ministry of Science and Higher Education (2012). However, the presented method is flexible and can accommodate all criteria at hand, including both quantitative and qualitative factors. No model is ideal and usually undergoes evolution as time passes. It is anticipated that CERU will be improving the model to evaluate academic entities at the national level. Using our approach to compute the weights is a time consuming but necessary exercise since it will benefit the entire country when the weights are computed (as opposed to arbitrary assignment). In particular, the success-index could improve the performance evaluation methods Franceschini et al (2012) in the evolved model.

## Footnotes

1 Following the official regulation, the authors understand that the scientific entity (sometimes also referred to as the scientific unit) means: a research unit within the university such as faculty or department, an independent research institute (national and international), and research units within The Polish Academy of Science.

2
http://www.bip.nauka.gov.pl/_gAllery/19/31/19319/poz._877

3 Please compare with Theorem 4 in Saaty (2008), where the importance weight assigned to every judge is identical.

4 Data used are real and taken from http://www.nauka.gov.pl. For the purposes of example the number of entities in the group SI1EA has been reduced from 44 to arbitrary selected 4.

5 A Polish Committee for Evaluation of Research Units; Polish acronym is KEJN

6 In Poland there are professor extraordinarius and professor ordinarius.

7 On the other hand, even if two rejected cases were taken into account their impact on the final result would be negligible.
